# Human Organ-on-a-Chip Microphysiological Systems to Model Musculoskeletal Pathologies and Accelerate Therapeutic Discovery

**DOI:** 10.3389/fbioe.2022.846230

**Published:** 2022-03-14

**Authors:** Raquel E. Ajalik, Rahul G. Alenchery, John S. Cognetti, Victor Z. Zhang, James L. McGrath, Benjamin L. Miller, Hani A. Awad

**Affiliations:** ^1^ Center for Musculoskeletal Research, University of Rochester, Rochester, NY, United States; ^2^ Department of Biomedical Engineering, University of Rochester, Rochester, NY, United States; ^3^ Department of Dermatology, University of Rochester, Rochester, NY, United States

**Keywords:** organ-on-chip, tissue-on-chip, microphysiologic systems, musculoskeletal, muscle, bone, cartilage, tendon and ligament

## Abstract

Human Microphysiological Systems (hMPS), otherwise known as organ- and tissue-on-a-chip models, are an emerging technology with the potential to replace *in vivo* animal studies with *in vitro* models that emulate human physiology at basic levels. hMPS platforms are designed to overcome limitations of two-dimensional (2D) cell culture systems by mimicking 3D tissue organization and microenvironmental cues that are physiologically and clinically relevant. Unlike animal studies, hMPS models can be configured for high content or high throughput screening in preclinical drug development. Applications in modeling acute and chronic injuries in the musculoskeletal system are slowly developing. However, the complexity and load bearing nature of musculoskeletal tissues and joints present unique challenges related to our limited understanding of disease mechanisms and the lack of consensus biomarkers to guide biological therapy development. With emphasis on examples of modeling musculoskeletal tissues, joints on chips, and organoids, this review highlights current trends of microphysiological systems technology. The review surveys state-of-the-art design and fabrication considerations inspired by lessons from bioreactors and biological variables emphasizing the role of induced pluripotent stem cells and genetic engineering in creating isogenic, patient-specific multicellular hMPS. The major challenges in modeling musculoskeletal tissues using hMPS chips are identified, including incorporating biological barriers, simulating joint compartments and heterogenous tissue interfaces, simulating immune interactions and inflammatory factors, simulating effects of *in vivo* loading, recording nociceptors responses as surrogates for pain outcomes, modeling the dynamic injury and healing responses by monitoring secreted proteins in real time, and creating arrayed formats for robotic high throughput screens. Overcoming these barriers will revolutionize musculoskeletal research by enabling physiologically relevant, predictive models of human tissues and joint diseases to accelerate and de-risk therapeutic discovery and translation to the clinic.

## 1 Introduction

Musculoskeletal conditions encompass a wide spectrum of pain or damage associated with muscle, bone, cartilage, tendon, ligament, joints, and nerves. Injuries to musculoskeletal tissues are the leading cause of disability worldwide and often limit mobility and restrict the patients’ ability to work or participate in recreational activities. As the mean age of the population increases, it is expected that the prevalence of musculoskeletal conditions and the associated socioeconomic burden will increase drastically in the coming decades (Sebbag et al., 2019). The most frequently reported musculoskeletal conditions in the US include arthritis, chronic joint pain, and back pain. Treatments typically prioritize pain-relief, including benzodiazepines, muscle relaxants, serotonin-norepinephrine reuptake inhibitors (SNRIs), non-steroidal anti-inflammatory drugs (NSAIDs), or combinations of those and other pain-relieving drugs (Hsu et al., 2019). While effective as palliative treatments, these are not disease-modifying or reparative drugs that address the molecular basis of the pathology. Therefore, there is a critical need for an improved understanding of the mechanisms of musculoskeletal pathologies to guide the development of drugs that resolve the underlying causes.

The process for drug and therapeutic discovery, development, and approval is arduous and costly. Discovery typically starts in academic or pharmaceutical laboratories. Unfortunately, many discoveries in academic laboratories fail to be reproducible or scaled up in pharmaceutical research and development laboratories. Leading drug candidates may be abandoned in the developmental “valley of death” because they are too slow or too costly to develop. And even well-funded efforts can fail in clinical trials despite promising preclinical findings. As a result, only ∼10% of the therapeutic development pipelines entering phase I clinical trials typically proceed to FDA approval. Without a technological breakthrough that reduces risk and front-end investments required for drug development, these significant barriers will continue to hamper development and translation efforts (Hay et al., 2014).

Less than 8% of active interventional clinical trials of musculoskeletal diseases in the United States involve disease-modifying biological therapies, including stem cell therapy, growth factors and platelet-rich-plasma trials. The shortage of biological therapies for musculoskeletal conditions, excluding arthritis, highlights several issues for the field to recognize and tackle. The American Academy of Orthopedic Surgeons (AAOS) Symposium in 2015 identified the root cause as the incomplete knowledge of musculoskeletal disease mechanisms and a lack of reliable biomarkers to inform clinical trials (LaPrade et al., 2016). In addition to frequent findings of poor efficacy, late-stage clinical trials fail from flawed study designs, inappropriate statistical endpoints, drug safety, or underpowered clinical trials resulting from patient dropouts and insufficient enrollment (Fogel, 2018). Although not all these factors can be controlled, there is an opportunity to address efficacy and safety assessment of therapeutic candidates earlier in the preclinical stages with better models of human musculoskeletal disease.

The level of evidence obtained from preclinical studies using animal models and *in vitro* culture systems is constrained by the limitations of these models. Animal models have been the cornerstone of translatable biomedical research over the past century. Despite their undeniable value in biomedical research, animal models have numerous limitations that unfortunately have contributed to the arduous and costly new therapy development process. Animal studies are intrinsically low throughput and do not accurately predict the drug’s effects and bioavailability in humans due to differences in pharmacokinetic and pharmacodynamic (PK/PD) responses. In addition, animal models used for biomedical studies are almost always inbred for research purposes and thus lack the genetic diversity of the human population. Lastly, studies of experimentally-induced acute or chronic musculoskeletal conditions do not use consensus models to allow uniformity in outcomes and valid interpretations of different experiments. Current *in vitro* culture models represent artificial, non-physiological conditions, mostly consisting of a single cell type or at best co-cultures of two cell types, to simulate the paracrine signaling between immune cells (e.g., macrophages) and mesenchymal cells (e.g., myocytes, fibroblasts, osteoblasts, osteocytes, and chondrocytes). Three dimensional (3D) scaffolds such as collagen are often used, but they are typically monocellular and not readily amenable to modeling the heterogeneity in a tissue. These systems are, therefore, inadequate to faithfully model treatment effects on acute or chronic musculoskeletal conditions or predict clinical outcomes.

**FIGURE 1:**
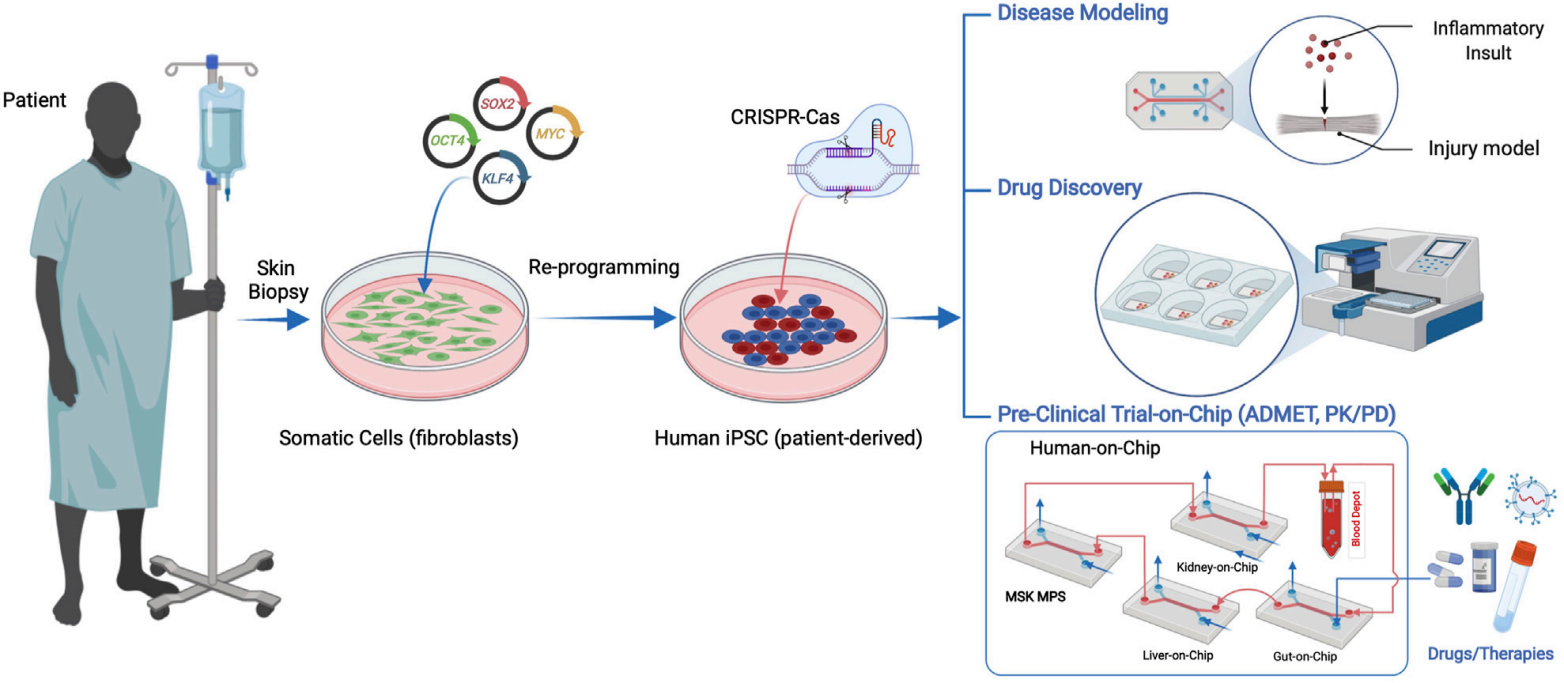
Human iPSCs can be derived from patient somatic cells procured through a minimally invasive tissue biopsy and reprogramming using pluripotency factors. hiPSC applications in microphysiological systems include disease models, drug discovery, and human preclinical trials on a chip, including ADMET (absorbance, distribution, metabolism, excretion, and toxicity) and pharmacokinetics and pharmacodynamics (PK/PD) studies.

Biomedical innovations such as induced pluripotent stem cells (iPSCs) from adult somatic cells (Takahashi and Yamanaka, 2006), CRISPR/CAS for gene editing (Jinek et al., 2012), and organ-on-a-chip (OoaC), also known as microphysiological systems (MPS), are ushering in an era where *in vitro* systems provide relevant, accessible, and flexible models of tissues and organs. MPS typically use microfluidic channels or compartments that model micro-scale units of multicellular tissues or organs (Bhatia and Ingber, 2014), tissue interfaces (Griep et al., 2013), and multi-organ systems (Sung et al., 2013). Ideally, MPS are allometrically scaled models of human tissues in their anatomical and physiological contexts. These technologies have various applications including disease modeling, drug discovery, toxicology screening, and personalized medicine (Esch et al., 2015). The integration of major tissues and organs in the human body in a single chip or connected chips to predict safety, efficacy and PK/PD of drug candidates in humans is one of the most exciting recent advances in the biomedical sciences (Figure 1). Therefore, MPS are a disruptive technology platform for evaluating safety and efficacy during the early stages of drug and therapeutic development and informing the planning and execution of clinical trials. This was recently demonstrated by a breakthrough study that used MPS of vascularized human kidney spheroids with integrated tissue-embedded microsensors for oxygen, glucose, lactate, and glutamine to provide real-time assessment of nephrotoxicity of immunosuppressive (cyclosporine) and anticancer (cisplatin) drugs. Importantly, the kidney-on-a-chip uncovered a previously unknown mechanism of injury involving glucose transport and predicted the protective effects of sodium-glucose cotransporter-2 (SGLT2) inhibitor (empagliflozin) against the nephrotoxicity induced by the immunosuppressive and anticancer drugs. The sensor-enabled kidney-on-a-chip prediction of safety and efficacy of the combination therapy was validated through retrospective analysis of a clinical study involving 247 patients receiving cyclosporine or cisplatin alone or in combination with the SGLT2 inhibitor empagliflozin (Cohen et al., 2021). Such works are paving the way for MPS technology to transform drug development and patient healthcare.

Despite the proliferation of these sophisticated chips to model various tissue and organ systems, scientists in the musculoskeletal field have been slow to develop and adopt them. For example, there has been significant progress in developing and validating MPS for major diseases associated with high mortality rates such as the heart (Marsano et al., 2016), lung (Huh et al., 2010), intestines (Bein et al., 2018), and liver (Raasch et al., 2019). Many of these systems are reported in the Microphysiology Systems Database (MPS-Db) developed by the University of Pittsburgh Drug Discovery Institute to aggregate and manage data from different laboratories and to provide reference and clinical data to evaluate and validate experimental MPS results (Gough et al., 2016). Currently, musculoskeletal models of bone, joint, and skeletal muscle account for <10% of the entries in the MPS-Db. Arguably, the slow adoption of MPS in studies of musculoskeletal diseases can be attributed to conceptual and practical challenges in modeling cell and extracellular matrix (ECM) interactions in the dynamic injury and healing processes, *in vivo* mechanical loading, incorporating vascularization and innervation, and recreating joints and sophisticated soft-to-hard tissue interfaces.

Therefore, this review outlines current tools and trends in the MPS field, citing examples of early applications to model musculoskeletal diseases. In addition to providing an overview of critical issues in MPS, the review discusses specific challenges that should be prioritized in future MPS models of musculoskeletal diseases to accelerate their adoption into the drug and therapeutic discovery pipeline and in virtual clinical trials.

## 2 From Bioreactors to Microphysiological Systems

**FIGURE 2:**
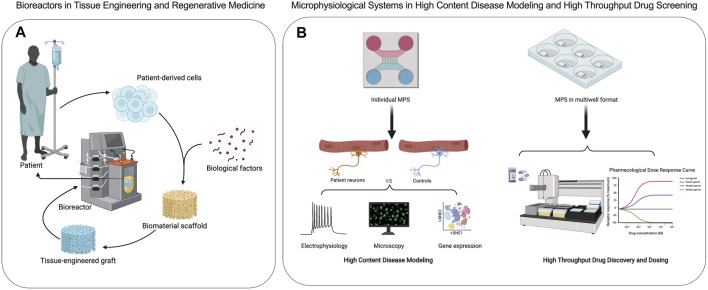
Applications of bioreactors and microphysiological systems are distinct. **(A)** Bioreactors are used to create engineered transplantable tissue grafts in tissue engineering and regenerative medicine. **(B)** Microphysiological systems are used in high content disease modeling and high throughput drug discovery and screening of efficacy and toxicity.

Bioreactors are closed cell and tissue culture systems in which the biochemical and biophysical environments of the culture are tightly regulated and monitored (Figure 2A). Traditionally bioreactors have been used as cell expansion systems for cell therapy, 3D engineered tissue training and maturation systems, and extracorporeal organ support devices (Ellis et al., 2005). MPS adopt many of the traditional bioreactor design principles, including perfusion, shear stimulation, or mechanical actuation (stretch or compression) on a much smaller scale with most applications focused on human disease research and drug screening (Figure 2B). Bioreactors and MPS manufacturing approaches are typically decoupled, where the tissue constructs and device components are manufactured separately and then assembled. This approach offers high flexibility in design through simulations and iterative prototypes before final manufacturing. For example, the design of a scaffold-free and perfused bioreactor can be optimized using computational fluid dynamic (CFD) simulations, allowing for tissue-specific designs to be optimized in silico. When tissue constructs with built-in microchannels are cultured in CFD-optimized bioreactors, effective nutrient perfusion and tissue maturation could be achieved over weeks of culture (Sego et al., 2020). This approach was utilized in the fabrication of autologous cartilage-bone grafts engineered for temporomandibular joint regeneration (TMJ). Both patient-specific geometry and scale were utilized to produce dual-perfusion bioreactors to match the patient’s geometry and cultivate mature constructs seeded with chondrogenic and osteogenic progenitors for weeks *in vitro* before demonstrating efficacy in TMJ reconstruction studies in large animals (Chen et al., 2020). The combination of computational simulation and experimental validation is commonplace in studies involving MPS as well (Aleman et al., 2019). In addition, bioreactors are commonly instrumented with sensors to monitor oxygen, glucose, lactate, and glutamine to provide real-time cell metabolism. The recent study from Cohen et al. (2021) suggested that tissue-embedded microsensors for oxygen, glucose, lactate, and glutamine in a kidney-on-a-chip MPS provide real-time assessment of cellular metabolism that led to the discovery of glucose transport as a nephrotoxicity mechanism associated with immunosuppressants and anti-cancer drugs (Cohen et al., 2021). Therefore, bioreactor design principles and technologies can be quite useful in informing design criteria and scaling down integrated sensors for MPS towards the goals of disease modeling and drug testing in clinically relevant contexts.

## 3 From Organoids to Organ-on-a-Chip

**FIGURE 3:**
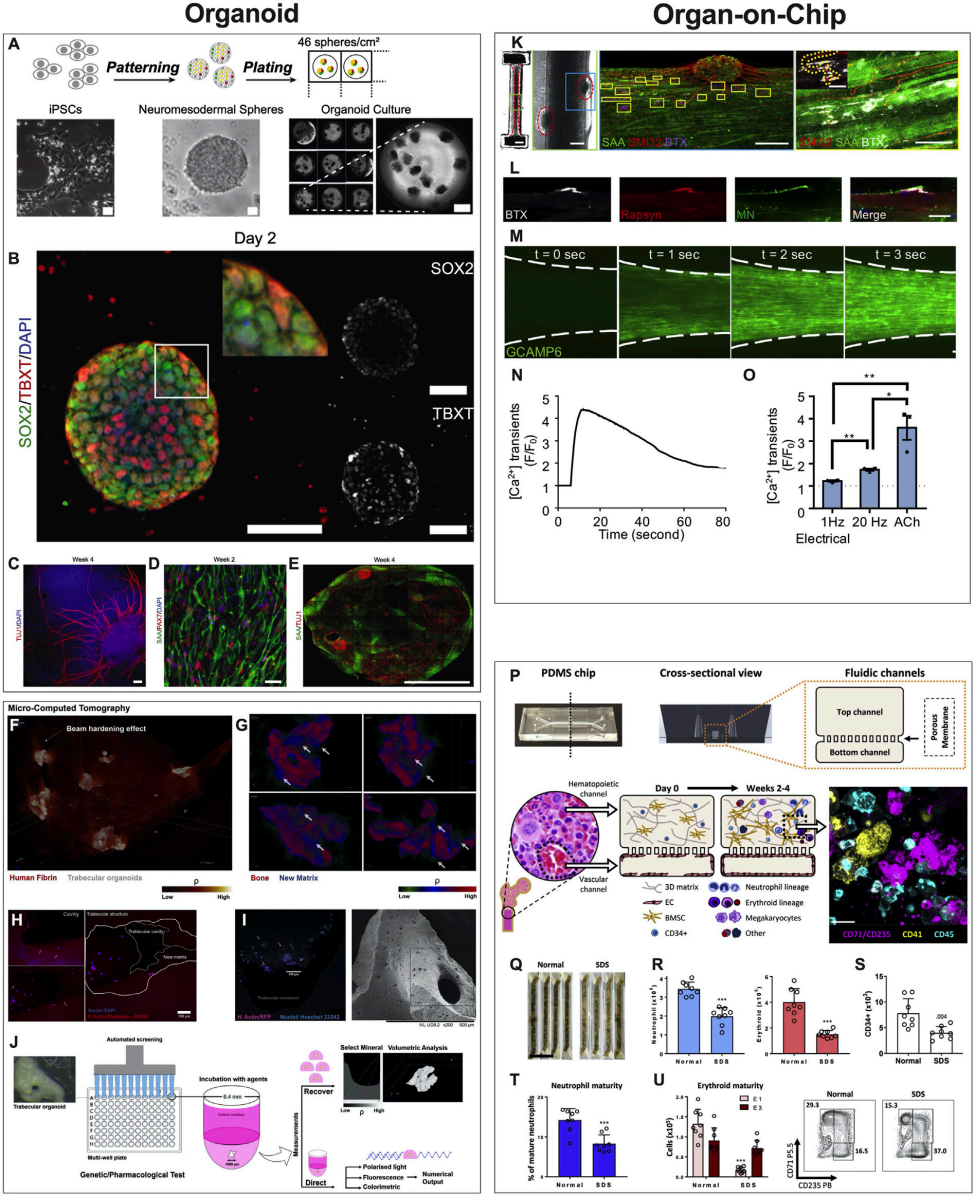
Examples of Organoids and Organ-on-a-Chip Microphysiological Systems. **(A–E)** Human sensorimotor organoid model uses iPSCs cultured in suspension and allowed to self-assemble into organoids and mature in culture over several weeks **(A)**, with immunostaining for neuronal stem cell (TUJ1), myogenic (TBXT, PAX7), neuromesodermal (SOX2/TBXT), and neurogenic (SOX2) transcription factors and the sarcomeric α-actinin (SAA) **(B–E)**. The platform was used to test several ALS traits and their effect on the NMJ, elucidating key events and attributes of motor neuron diseases. [Reproduced from (Pereira et al., 2021) with permission]. **(F–J)** Trabecular bone in fibrin gel organoids **(F)** demonstrating bone remodeling *in vitro* **(G)** via the coupled activities of osteoblasts **(H)** and osteoclasts **(I)**. This human trabecular bone organoid allows for detailed morphologic and resorption events to be studied and chemically characterized **(J)**, including investigating the effects of microgravity on bone loss. [Reproduced from (Iordachescu et al., 2021) with permission]. **(K–O)** 3D neuromuscular co-culture in an organ-on-a-chip augments AChR signaling. A representative 3D skeletal muscle-motor neuron (MN) co-culture at 2 weeks **(K)**. Neuromuscular tissue outlined with red dashed line in left panel. Representative confocal image of a 2-week old neuromuscular co-culture immunostained for sarcomeric α-actinin (SAA; green), α-bungarotoxin (BTX; magenta), and neurofilament heavy SMI-32 (red). A neuromuscular co-culture immunostained on Day 10 of differentiation for Rapsyn (red), bungarotoxin (BTX, white), and counter stained with Hoechst 33342 (blue). **(L)** Epifluorescence images of a GCaMP6-labeled transduced 3D muscle tissue to visualize muscle fiber calcium transients at time-points before (t = 0 s) and after (t = 1, 2, and 3 s) ACh stimulation. **(M)** Time course of GCaMP6 reporter fluorescence following ACh-induced stimulation of a representative 3D muscle tissue. **(N)** Quantification of GCaMP6 signal after 3D skeletal muscle tissue low (1 Hz) or high (20 Hz) electrical stimulation, or ACh biochemical stimulation, and relative to phosphate buffered saline treated control tissues (dotted line). **(O)** [Reproduced from (Afshar Bakooshli et al., 2019) with permission]. **(P–U)** The design of the human bone marrow on chip recapitulates human bone marrow histology through a vascular layer in contact with bone marrow derived mesenchymal stromal cells embedded in an extracellular matrix with immune cell progenitors over 2–4 weeks. **(P)** BM Chips seeded with CD34^+^ cells from normal donors versus SDS patients at 2 weeks of culture. **(Q)** Neutrophil [**(R)**, left)], erythroid [**(R)**, right], and CD34^+^ **(S)** cell numbers were quantified by flow cytometry. Percentages of neutrophils with a mature CD16hi surface phenotype in control versus SDS BM Chips were quantified by flow cytometry **(T)**. Number of erythroid cells at different maturation states (left) and representative flow plots (right) depicting the percentages of the erythroid subpopulations (E1: immature, E3: mature), as quantified by flow cytometry **(U)**. (Reproduced from (Chou et al., 2020) with permission).

Organoids are defined as 3D multicellular *in vitro* tissue constructs that mimic corresponding *in vivo* organs, such that they can be used to study aspects of that organ in the tissue culture system (De Souza, 2018). In general, they lack preconceived structure and architecture provided through intentional design of the micro tissue. Despite this, organoids can acquire the native organ’s 3D complexity and functionality *in vivo* based on cell-cell communication, spatial cues and gradients (Rossi et al., 2018). Organoids can be used in MPS applications involving disease models, drug discovery and testing, and regenerative medicine. For example, human iPSCs derived from ALS patients were used to create functional sensorimotor organoids (neuromuscular junctions (NMJs)) to probe how distinct ALS variants may impair skeletal muscles and motor neurons at the level of the NMJ (Figures 3A–E) (Pereira et al., 2021). Other examples include trabecular bone organoids, formed by seeding primary osteoblasts and osteoclasts onto femoral head micro-trabeculae, which are then encapsulated in fibrin spheroids and cultured to model a pathological bone mass loss due to simulated microgravity (Figures 3F–J) (Iordachescu et al., 2021). Additional examples of organoid based MPS of musculoskeletal tissues are listed in Table 1. These examples provide a strong case for organoid-based microphysiological disease models for therapeutic screening. Advantages of using organoids as MPS include the close mimicry to embryonic cell assembly and tissue growth *in vivo* and the small size of spherical organoids that represent fully functioning microphysiological units. Disadvantages include the high variability in self-assembling cell clusters and difficulty in controlling the culture conditions, including those required for reproducible differentiation.

**TABLE 1:** Examples of organoid models of musculoskeletal tissues and organs.

Organ/tissue represented	Disease/disorder or application	Treatment tested	Cell type used	References
Skeletal cartilage	Pharmacological and environmental toxicity and Shwachman-diamond syndrome (SDS)		Adult human bone marrow-derived mesenchymal progenitor cells (hBM-MPCs)	Pirosa et al. 2019
Cartilage and bone	Osteoarthritis	Scaffold-mediated lentiviral gene delivery of dox-inducible cytokine inhibitors and growth factors	Human MSCs	Rowland et al. 2018
Trabecular Bone	Degenerative effects induced by low-shear mechanical stimulation	—	Primary human osteoblasts and Primary human osteoclast precursors	Iordachescu et al. 2021
Trabecular Bone	Regulation of bone remodeling	—	Murine osteogenic cells	Park et al. 2021
Neuromuscular Junction	Amyotropic lateral sclerosis (ALS)	—	iPSCs and ALS mutated isogenic iPSC lines	Pereira et al. 2021
Neuromuscular Trunk	Neuromuscular degenerative diseases	—	Human pluripotent stem cells and iPSCs	Faustino Martins et al. 2020

MPS are most commonly synonymous with organ-on-a-chip (OoaC), microdevices engineered to contain patterned cells and ECM to model tissue and organ structure and function at the micro-scale. The defining characteristics of OoaC include recreating the 3D arrangement of tissues on the platform, patterning multiple organotypic cells in a physiologically relevant context, and simulating biochemical (signals) and biophysical (forces) cues relevant to the modeled tissue or organ. For example, the effectiveness of co-culturing 3D human skeletal muscle fiber tissues with human iPSC-derived motor neurons to study the NMJ depends on whether the model is 2D or 3D. When 3D NMJ-on-chip models were compared to 2D culture conditions, they demonstrated functional superiority in fiber maturation and Acetylcholine receptors (AChR) clustering that affected the electrophysiological responses, strongly suggesting that 3D NMJ-on-chip is a powerful model to study adult human sensorimotor synaptogenesis and NMJ disease *in vitro* (Figures 3K–O) (Afshar Bakooshli et al., 2019).

In addition, the periarterial, perisinusoidal, mesenchymal, and osteoblastic hematopoietic niches in the bone marrow (BM) *in vivo* have been modeled in a two-channel vascularized BM-on-chip platform. The BM channel was simulated using a fibrin hydrogel in which CD34^+^ cells and marrow-derived stromal cells were co-cultured, whereas the vascular channel, separated from the channel using a porous elastic membrane, was lined with endothelial cells cultured under media flow (Figures 3P–U). The BM-on-chip device reproduced key features in the BM hematopoietic stem cell niche and simulated expected pathologies in hematopoiesis when constructed using cells from SDS (Shwachman–Diamond syndrome) patients (Chou et al., 2020). Additional examples of OoaCs based MPS of musculoskeletal tissues are listed in Table 2. These examples demonstrate the advantages of OoaC microdevices, which provide physiologically relevant, human-specific alternatives to animal testing for the study of disease pathophysiology, and the ability to recapitulate clinically relevant disease states by engineering features of organ architecture and physiology at the microscale. Disadvantages include the simplistic nature of these disease models. Regardless, the common aphorism stating that “*all models are wrong, but some are useful*” applies here. The level of complexity needed in creating these models must balance a real value that otherwise could not be attained from a simpler model.

**TABLE 2:** Examples of tissue-on-a-chip models of musculoskeletal tissues and organs.

Organ/tissue represented	Disease/disorder or application	Treatment tested	Cell type used	References
Neuromuscular Junction	Motor neuron disease (MND)	—	Human embryonic stem cells (hESC), human iPSC-derived MNs (ESCs and iPSCs as healthy control), or human iPSC-derived MNs from patients with NMD, in combination with human iPSC derived skeletal muscle cells	Osaki et al. 2020
Neuromuscular Junction	Myasthenia gravis	—	Human primary fibroblasts, human PSC motor neurons	Afshar Bakooshli et al. 2019
Muscle	Acute oxidative injury and cancer cahexia	—	Human MSCs (Lonza), human skeletal myoblasts (hSKMB; Lonza) A549 lung adenocarcinoma spheroids, human lung fibroblasts, THP-1-derived macrophages	Mondrinos et al. 2021
Skeletal muscle	Oxygen deficits in skeletal muscle during exercise	—	Primary human myoblasts	Davis et al. 2019
Skeletal muscle	Hypertrophy	—	Primary human myoblasts	Khodabukus et al. 2019
Skeletal and smooth muscle	Duchenne muscular dystrophy (DMD)	—	Healthy & DMD derived human muscle myoblasts	Nesmith et al. 2016
Skeletal muscle	—	Biohybrid valveless pump-bot powered by “living” engineered skeletal muscle	C2C12 mouse skeletal myoblasts	Li et al. 2019
Skeletal muscle	Screening platform and *in vitro* muscle injury model	Cardiotoxin	C2C12 mouse murine myoblast cell line	Agrawal et al. 2017
Cartilage and bone junction	Osteoarthritis	Celecoxib	iPSC-derived mesenchymal progenitor cells (iMPCs)	Lin et al. 2019
Articular cartilage	Osteoarthritis	Triamcinolone steroid treatment	Primary equine chondrocytes	Rosser et al. 2019
Articular cartilage	Osteoarthritis	Interleukin-1 receptor antagonist (IL-1Ra) and rapamycin	Primary human articular chondrocytes (hACs)	Occhetta et al. 2019
Articular joint	Osteoarthritis	RS-504393 (CCR2 antagonist) and Cenicriviroc (CCR2/CCR5 antagonist)	Primary synovial fibroblasts, articular chondrocytes, GFP-HUVECs, PBMC derived monocytes, patient synovial fluid	Mondadori et al. 2021
Bone marrow niche	Interaction of infused HSPC, lymphoma and leukemic cells	—	Bone marrow mononuclear cells (BMNC), Stro-1+ MSC	Aleman et al. 2019
Hematopoietic microenvironment	—	—	HUVECs, Stromal fibroblast cell lines (HS5-GFP & HS27a-GFP), Peripheral blood mononuclear cells (PBMCs), Mesenchymal stem cells (MSCs)	Kotha et al. 2018
Bone perivascular niche	Breast cancer cell colonization into bone		Endothelial cells, bone marrow MSCs and MDA-MB-231/GFP or MDA-MB-231/Luc cells	Marturano-Kruik et al. 2018
Bone marrow	Model of hematopoietic response to drug exposure, ionizing radiation, and genetic mutation	—	Human CD34 cells, patient derived Bone marrow stromal cells, primary human-derived bone marrow mononuclear cells	Chou et al. 2020
Bone	Breast cancer	—	Murine calvaria preosteoblasts (MC3T3-E1) and human breast cancer cell lines MDA-MB-231^GFP^ and its metastatic suppressed variant MDA-MB-231 ^GFP^	Hao et al. 2018

## 4 Considerations for Building Musculoskeletal Microphysiological Systems

### 4.1 Cell Sources

There are various cell sources for MPS, which include primary cells, cell lines, stem cells, and iPSCs. Each source has advantages and disadvantages, which must be factored in their utilization in MPS applications. Arguably, these cells must be derived from human sources for the MPS to have their highest impact.

#### 4.1.1 Primary Cells

Primary or somatic cells are directly isolated from living tissue and ideally suited to model the tissue from which they are extracted. In many tissues, primary cell isolation retrieves heterogeneous populations, including tissue-resident stem cells (Alberts et al., 2002). This necessitates protocols to purify and enrich the desired cell type based on a variety of sorting criteria and technologies. Furthermore, dissociating the different subpopulations of cells from the tissue results in a loss of important spatial cues and gradients, leading to dedifferentiation (Shoshani and Zipori, 2015). To maintain the differentiated cell phenotype, specific culture media, culture conditions, and matrix requirements must be empirically optimized. Despite their obvious advantages in performing their differentiated functions and secreting tissue-specific molecules, primary cells have limitations. Primary cells experience stress-induced senescence after multiple passages and endure replicative aging and contact inhibition that limit their proliferation *in vitro* (Sherr and DePinho, 2000). Although commonly seen in aging tissues, replicative senescence *in vitro* poses significant challenges in modeling diseases, especially when senescence is not a feature of the disease. Furthermore, the availability of primary cells is limited because their harvest is typically invasive and can result in significant donor site morbidities. This is a serious limitation in modeling or integrating different types of patient-specific cells in MPS.

#### 4.1.2 Immortalized Cell Lines

Human primary cells undergo a limited number of cell divisions (40–60) in culture before they reach senescence and lose their ability to divide. This loss of proliferative ability is attributed to reduced telomerase activity at high passage numbers (Hayflick and Moorhead, 1961). Therefore, immortalized cell lines, created using techniques such as human telomerase reverse transcriptase (hTERT), can overcome the Hayflick limit of primary cells. Cell lines can undergo unlimited divisions in culture at low cost, making them readily available from several commercial vendors and widely used across numerous laboratories worldwide. These properties afford them desirable consistency and reproducibility, making them a popular choice for basic research to study the molecular mechanisms of human disease in academic laboratories. They are also used in industrial production of recombinant proteins and vaccines and for drug cytotoxicity testing. Unfortunately, these cell types exhibit significant chromosomal and genetic abnormalities, which limit their ability to reproduce normal cell behavior *in vitro* (Lorsch et al., 2014). These drawbacks do not always outweigh the simplicity of using immortalized cell lines, particularly during MPS development where design iteration and testing does not require physiologically accurate cells. Cell lines should be replaced by more representative cells however, in the translational application of MPS models.

#### 4.1.3 Stem Cells

Stem cells are perhaps the single most important discovery in regenerative medicine. They possess properties that can theoretically correct pathological changes caused by disease or injury. However, stem cells and their progenies can also be used as primary components of personalized (patient specific) MPS models of human disease. By definition, stem cells are characterized by the ability to self-renew indefinitely by symmetric or asymmetric cell division while maintaining an undifferentiated state and the ability to differentiate into the various fates of specialized cell types under the right chemical and biological cues. This latter property refers to the regenerative potency of stem cells. While the nomenclature is generally not uniformly used, pluripotent stem cells can differentiate into cells from any of the three embryonic germ layers: the ectoderm, the mesoderm, and the endoderm. First successfully isolated from the inner cell mass of the blastocyst by James Thompson in 1998 (Thomson et al., 1998), human embryonic stem cells (hESC) are demonstrably pluripotent, making them promising therapies in regenerative medicine and indispensable tools for basic research, drug discovery and testing. Therefore, they represent an important cell source for MPS models of human disease. However, the use of hESC is contentious due to ethical concerns related to their embryonic source, which restricted federal funding for continued generation of new hESC colonies. Moreover, early colonies of hESC have been generated using murine fibroblast feeder layers and animal sera, making them potentially unsuitable for clinical applications of regenerative medicine. As so, the derivation of differentiated cells from high passage hESC, some of which are now greater than 20 years-old, should be carefully assessed to ensure the cells have not accumulated abnormal traits. Alternative sourcing of hESC can be achieved through somatic cell nuclear transfer (SCNT or cloning) but this also raises ethical concerns about the unhinged use of this technology to clone human fetuses. In addition, the empirical protocols to generate and maintain hESC in their undifferentiated state are inefficient and could lead to spontaneous uncontrolled differentiation events that must be monitored and eliminated carefully.

Alternative sources of stem cells include extraembryonic fetal tissues such as the placenta and the umbilical cord (Wharton’s jelly). In addition, stem cells have been identified in specialized compartments or niches in numerous tissues that retain a moderate level of regenerative abilities throughout postnatal and adult life. These adult stem cells are multipotent, such that they can differentiate into different cell types that make the tissue or related tissues from the same embryonic germ layers. As with primary cells, the invasive isolation of human adult stem cells is associated with tissue injury and donor site morbidity that constrain their applications in MPS models.

However, bone marrow, which harbors hematopoietic stem cells (HSC) and mesenchymal stromal (also called stem) cells (MSC), is a replenishable, readily accessible source of adult stem cells with minimal morbidity. HSC give rise to myeloid and lymphoid progenies of blood and immune cells through well-characterized steps of differentiation, which can be replicated *in vitro* (Orkin and Zon, 2008). As such, marrow-derived HSC represent an important source of cells for MPS models incorporating interactions with immune cells. Marrow-derived mesenchymal cells were first identified by Friedenstein due to their clonogenic (colony forming) replicative properties and their propensity for osteogenic differentiation *in vitro* and ectopic bone formation *in vivo* (Friedenstein et al., 1987). Caplan later coined the term mesenchymal stem cells and significantly advanced the concept of mesengenesis, the differentiation of musculoskeletal progenies from MSC (Caplan, 1994). Protocols for isolating and characterizing human MSC and demonstrating the multilineage potential of MSC were later developed (Pittenger et al., 1999). Numerous studies have since demonstrated the ability of MSC to differentiate into musculoskeletal cells to be used in cell therapy and tissue engineering, including osteoblasts, chondrocytes, myocytes, tenocytes, adipocytes, and endothelial cells [reviewed in (Andrzejewska et al., 2019)]. Stem cells were also isolated from other tissues such as adipose-derived stem cells (ASC), which display comparable multilineage differentiation potential to marrow-derived MSC. Both MSC and ASC, as well as other tissue-derived adult stem cells, are now believed to be pericytes in vascular niches of these tissues (Crisan et al., 2008). And while the current paradigm in regenerative medicine has now shifted to emphasize the immunomodulatory and trophic phenotypes of adult stem cells with respect to their therapeutic potential (Caplan, 2017), their utility in MPS models of musculoskeletal diseases still requires efficient and reproducible multilineage differentiation protocols. Therefore, human marrow-derived HSC and MSC can potentially be useful tools in constructing isogenic vascularized tissue MPS models of musculoskeletal diseases.

#### 4.1.4 Induced Pluripotent Stem Cells

The Noble prize discovery that somatic cells can be reprogrammed to turn back the clock and induce a pluripotent stem cell-like state opens limitless possibilities for applications in MPS models. iPSCs were originally derived from murine embryonic and adult fibroblasts through viral transfection with *Oct3/4*, *Klf4*, *Sox2*, and *c-Myc* pluripotency factors (Takahashi and Yamanaka, 2006). This discovery was later translated to generate human iPSCs through transfection of hESC–derived somatic cells, primary fetal tissues (lung, skin), and neonatal and adult dermal fibroblasts with the same or similar factors (*OCT4*, *SOX2*, *NANOG*, *LIN28, or KLF4, MYC*) (Yu et al., 2007). Today, numerous well characterized human iPSCs can be purchased from commercial vendors and cell banks or generated on demand from adult fibroblasts in academic laboratories and through commercial services. Numerous studies indicate human iPSCs bear a remarkably comparable pluripotency to hESCs. Therefore, hiPSCs hold great potential in cell and gene therapy to address genetic and degenerative diseases. Techniques such as *ex vivo* gene therapy or CRISPR/Cas9 can correct genetic mutations associated with various diseases in patient-derived iPSC progenies, which can then be used in autologous cell and gene therapies. Personalized, autologous iPSC-based therapies circumvent triggering an immune response and subsequent graft rejection. Additionally, human iPSCs hold great potential in MPS-based disease models for drug discovery and toxicity testing (Bellin et al., 2012).

In particular, the use of human iPSC-derived cells from an autologous donor overcomes the previously intractable problem of creating interconnected, isogenic MPS models of multiple tissues or organ, paving the way towards clinically relevant human-on-chip models (Figure 1). Therefore, human iPSC technology is highly valuable in MPS models of musculoskeletal conditions, especially for composite tissues that require incorporating different cell types such as muscle-tendon (myotendinous junctions) and tendon/ligament-bone interface (entheses), osteochondral tissues, vascularized and innervated muscle and bone tissues or composites thereof.

Despite their undeniable potential for MPS applications, the use of human iPSC in modeling human diseases has limitations. The relationship between the genome and epigenome has broadened the understanding of the types of molecular events that cause human disease. Current strategies for iPSC generation/regeneration of isogenic tissues eliminates this epigenetic memory from donor cells while maintaining the patient’s intact genome. Although human iPSC are effective for modeling purely genetic disease (e.g., Amyotrophic Lateral Sclerosis or Duchenne muscular dystrophy), they have limitations in modeling diseases stemming from both genetic and epigenetic factors. They also have other challenges in modeling important biological variables that maybe systemic rather than purely cellular, including sex and age.

**FIGURE 4:**
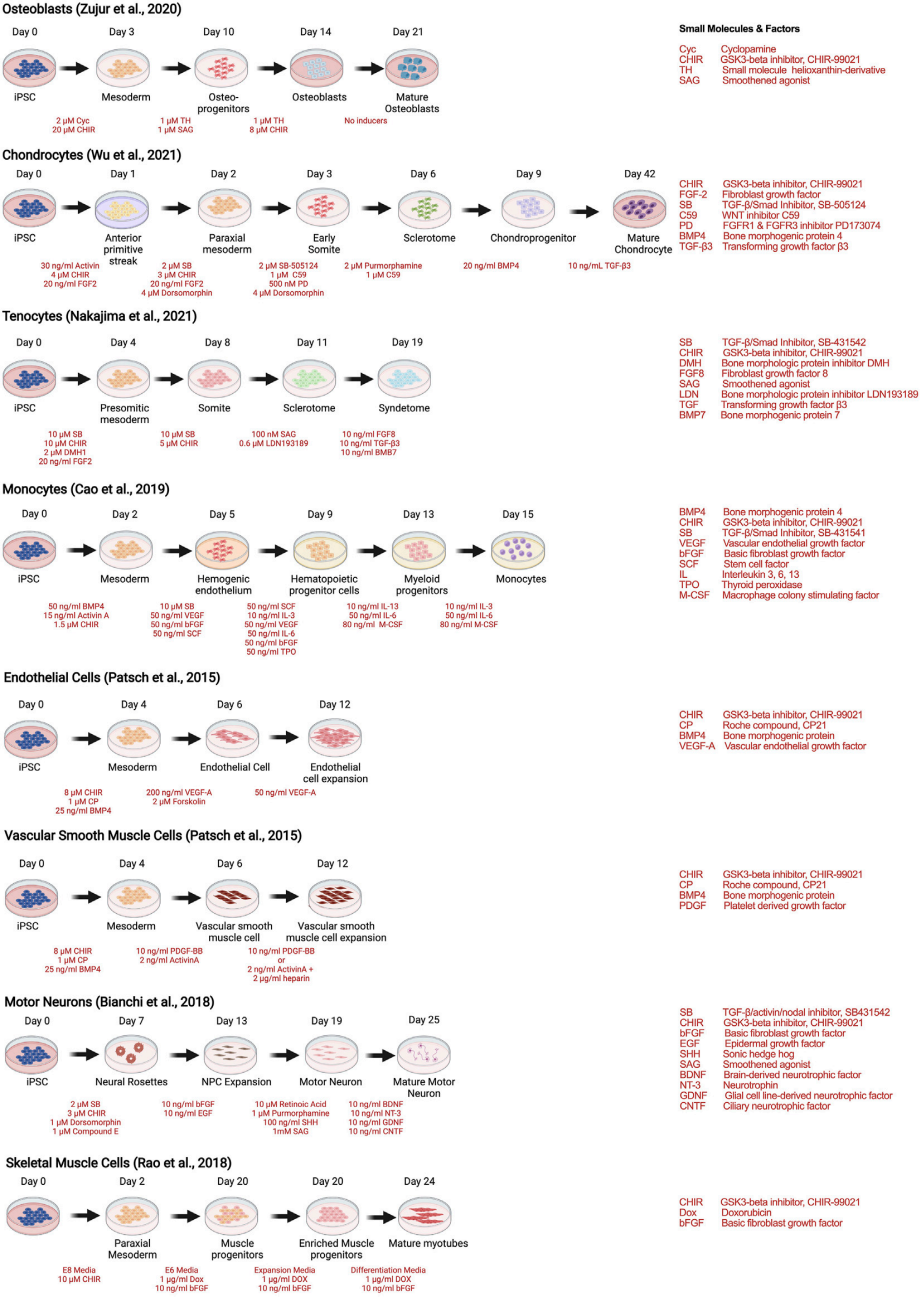
Strategies to derive musculoskeletal cells from human iPSCs through stepwise differentiation.

As with stem cells, the utility of human iPSCs in MPS models of musculoskeletal diseases requires efficient and reproducible multilineage differentiation protocols. Strategies to generate human iPSC musculoskeletal derivatives have been described in numerous publications, including osteoblasts (Zujur et al., 2020), tenocytes (Nakajima et al., 2021), chondrocytes (Wu et al., 2021), skeletal muscle (Rao et al., 2018), motor neurons (Bianchi et al., 2018), endothelial cells and vascular smooth muscle cells (Patsch et al., 2015), and monocytes and their progenies (macrophages and dendritic cells) (Cao et al., 2019), depicted as examples of recent protocols in Figure 4.

### 4.2 Extracellular Matrix Biomaterials

Polymeric hydrogels are widely used in MPS platforms as they resemble the macromolecular ECM of many tissues and organs, providing proper cellular architecture, support, and function. The three main categories of hydrogels include natural, synthetic, and hybrid materials. Animal sourced natural hydrogels such as collagen and fibrin are biocompatible and provide native cell-binding ligands and biochemical properties present in native tissues. On the other hand, the process of deriving natural hydrogels from animals results in limited mechanical strengths, long-term storage instabilities, and batch differences, reviewed in (Terrell et al., 2020). Alternatively, synthetic hydrogels provide more control over mechanical strength and batch differences as they are chemically synthesized from precursor molecules. However, synthetic hydrogels often require additional chemical modifications to promote cell adherence and viability (Cruz-Acuña and García, 2017). Often, hybrid hydrogels are synthesized from biological macromolecules such as hyaluronic acid, which can be combined with synthetic hydrogels or chemically modified using acrylation reactions to enable photo crosslinking upon the addition of photo initiators and exposure to light (Jiang et al., 2020).

Aside from hydrogels, ECM derived biomaterials also include decellularized scaffolds which provide a native environment for tissue engineering, electrospun fibrous scaffolds providing nano-microscale fibrous structures with interconnecting pores, and 3D-printed materials fabricated to fit the desired geometry. Materials for these approaches can be native (tissue-derived), synthetic, or hybrid. The primary advantages of these approaches are the controlled architecture acquired through scaffolding and high-definition fabrication techniques achieved with electrospinning and 3D-printing. Limitations of these approaches include the difficulty in recreating cell-ECM interactions *in vivo* and biomechanically matching the tissue of interest with a biomaterial scaffold. Although not thoroughly covered here, others have extensively reviewed the biomaterials and fabrication methods which are key components in successful representation of musculoskeletal disease models (Cunniffe et al., 2019; Li et al., 2021).

### 4.3 Fabrication Materials in MPS Devices

Beyond the conceptual design of the MPS, practical considerations influence the choice of materials used in device fabrication. The most commonly used fabrication materials for MPS devices are elastomers and rigid polymers. The choice of the fabrication materials affects manufacturability, assembly flexibility, maintaining sterility, incorporating precise physical stimuli (e.g., stretch and flow-induced shear), molecular adsorption and absorption, and longitudinal monitoring of outcomes through live microscopy or integrated sensors.

#### 4.3.1 Elastomers

Elastomers are cross-linked polymers with weakly entangled chains. Due to their low elastic moduli and weak intermolecular forces, they easily deform (stretch or compress) and experience high strains without failure when an external force is exerted but return to their undeformed state when the force is withdrawn. Polydimethylsiloxane (PDMS) is an elastomer that has been widely adopted in the microfluidics community for its versatility, biocompatibility, permeability, and low cost. PDMS has been crucial in the early work of MPS and remains a prodigious material, with most devices still utilizing PDMS as their primary structural component. Despite its popularity, some limitations of PDMS include incompatibility with inorganic solvents, absorption of small hydrophobic molecules, adsorption of biomolecules, and gas permeability that lead to changes in concentrations of solutions over time (Ren et al., 2013). The multilayer soft lithographic fabrication method of PDMS is widely implemented in the MPS field yet is inadequate for industrial and high-throughput applications, both of which will be necessary if MPS are to be adopted into the drug development workflows. As an alternative, thermoplastic elastomers (TPE) offer thermoforming processing while maintaining low cost, optical transparency, biocompatibility, and flexibility comparable to PDMS. Flexible elastomers such as tetrafluoroethylene-propylene (FEPM), poly (polyol sebacate), and poly (esteramide) have been developed as PDMS alternatives. For example, Sano et al. (2019) modeled the endothelial-epithelial interface in a MPS by combining two FEPM microchannels separated by a collagen membrane that permits fluid flow through the channels and mechanical strain through vacuum chambers (Sano et al., 2019). Such innovations are highly practical as many MPS currently prototyped with PDMS can be re-designed with such materials. Reducing absorption of small hydrophobic drugs signified the potential of FEPM as an alternative to PDMS for drug discovery, with other materials offering similar performance such as poly [octamethylene maleate (anhydride) citrate) (POMaC) (Zhao et al., 2019]. Nonetheless, these alternative elastomers do not fully eliminate small molecule absorption in MPS and research into novel nonabsorbent elastomers with optical transparency, flexibility, and ease of fabrication continues. For the musculoskeletal field, elastomers have the highest impact as many of the tissues require bulk-tissue actuation (i.e., tendon stretching, cartilage compression) that can be hard to achieve without flexible materials.

#### 4.3.2 Rigid Polymers

Rigid thermoplastic polymers such as Polystyrene (PS), Poly (methyl methacrylate) (PMMA), Polyurethane, Teflon, and PEGDA are high strength, relatively inflexible, low cost, light weight, optically transparent, and biocompatible (low monomer leaching) materials widely used in fabricating MPS devices. These rigid thermoplastics can be reshaped multiple times by reheating, which is advantageous for molding and bonding. These materials can be fabricated through silicon master molds, reactive ion etching (REI), injection molding and hot embossing. Therefore, thermoplastic components of MPS devices can have high upfront production and development costs not feasible for prototyping but suitable for large batch manufacturing. As an alternative, rapid prototyping methods include CNC micromilling techniques and 3D printing to allow for quick, low-cost fabrication at the benchtop.

Generally, rigid polymers show improved solvent compatibility compared to PDMS including some resistance to alcohols. However, they are incompatible with organic solvents such as ketones and hydrocarbons. Their low gas permeability makes them unsuitable for long-term and static cell studies in sealed microchannels and microchambers. These environments limit gas permeance which can be lethal to cells, particularly in incubators where CO_2_ exchange is necessary for buffering the cell media and can accumulate in impermeable, static platforms. Yet, it can be optimal when using media with premixed gases to monitor dissolved oxygen consumption and pH levels in the MPS environment for example (Müller et al., 2021). The advantages of impermeability were observed in two studies where PMMA and PS were compared to PDMS devices and demonstrated more reliable results in drug toxicity and effectiveness screening. In both cases, the rigid thermoplastic polymer MPS results resembled *in vivo* findings with respect to cytotoxicity and drug effectiveness compared to the PDMS MPS, which likely experienced dosage changes overtime due to protein absorption by the PDMS surfaces (Nguyen et al., 2019). Although optimal for microscopy and PK/PD studies, the rigidity of these materials makes it difficult for actuation to be incorporated. Therefore, hybrid MPS devices are common to take advantage of elastomeric and rigid polymer properties.

## 5 Challenges for Musculoskeletal MPS

The implementation of intentional design strategy is key to successful MPS application. This requires that MPS platforms are designed to: 1) model the disease within the context of the tissue’s physiological and functional parameters, 2) enable longitudinal and endpoint assays, and 3) accommodate the skills, resources, and objectives of the end user. Physiological and functional parameters include gradients, heterogenous interfaces, biological barriers, mechanical or electrical stimulation, fluid flow, and interconnectivity with different tissue or organ chips. Longitudinal assays include brightfield and fluorescent microscopy, multiplex sensing of secreted proteins, while destructive endpoint assays include histology, immunohistochemistry, and q-PCR. End users could be trainees in academic laboratories pursuing high content data to uncover mechanisms of disease, drug discovery scientists in a pharmaceutical R&D facility pursuing high throughput data to identify hits that could be developed as drugs or biologics, or clinical trial technicians seeking proof of safety or efficacy of a drug candidate.

In addition to the challenges of creating clinically-faithful disease models with biomarkers that capture the dynamic nature of acute or chronic pathologies, tissues and joints in the musculoskeletal system, there are various specialized features that require engineering innovations to model them on MPS. For example, the migration of innate and adaptive immune cells from the bone marrow into the vasculature, the infiltration of platelets, neutrophils, macrophages, and various immune cells to sites of tissue injury, and cancer metastases growth underscore the importance of engineering permeable vascular barriers. Biological interfaces and ECM gradients, such as the myotendinous junction and the enthesis are critical not only for mechanical function but also for cellular functions and signaling, and there are several engineering approaches to engineer gradients that would need to be scaled down for MPS. Directed motor neuron terminal attachment to highly organized muscle fibers is another example of the intricate microarchitectural engineering that would be required to model innervated tissues (Rodríguez Cruz et al., 2020). Furthermore, sophisticated joint-on-chip designs must create closed compartments of joint cartilage and the underlying subchondral bone, synovial fluid, and vascularized synovial capsular tissues with articular motion to simulate nutrient transport, lubrication, *in vivo* loading, and inflammation. Importantly, engineering outcomes of pain indicators in MPS is critical to designing clinical trials-on-chip since pain is one of the most significant patient-reported outcomes in the clinic.

The following sections discuss seven challenges that should be prioritized in future musculoskeletal MPS platforms to increase the predictive power of these models in disease research and drug discovery areas. These challenges include engineering biological barriers, engineering heterogenous tissue interfaces, incorporation of immune cells and inflammatory factors, biomechanical actuation and loading, incorporating surrogate measurements for pain, integrating inline sensors for real-time monitoring of dynamic processes, and creating arrayed formats for high throughput screening.

### 5.1 Engineered Biological Barriers

**FIGURE 5:**
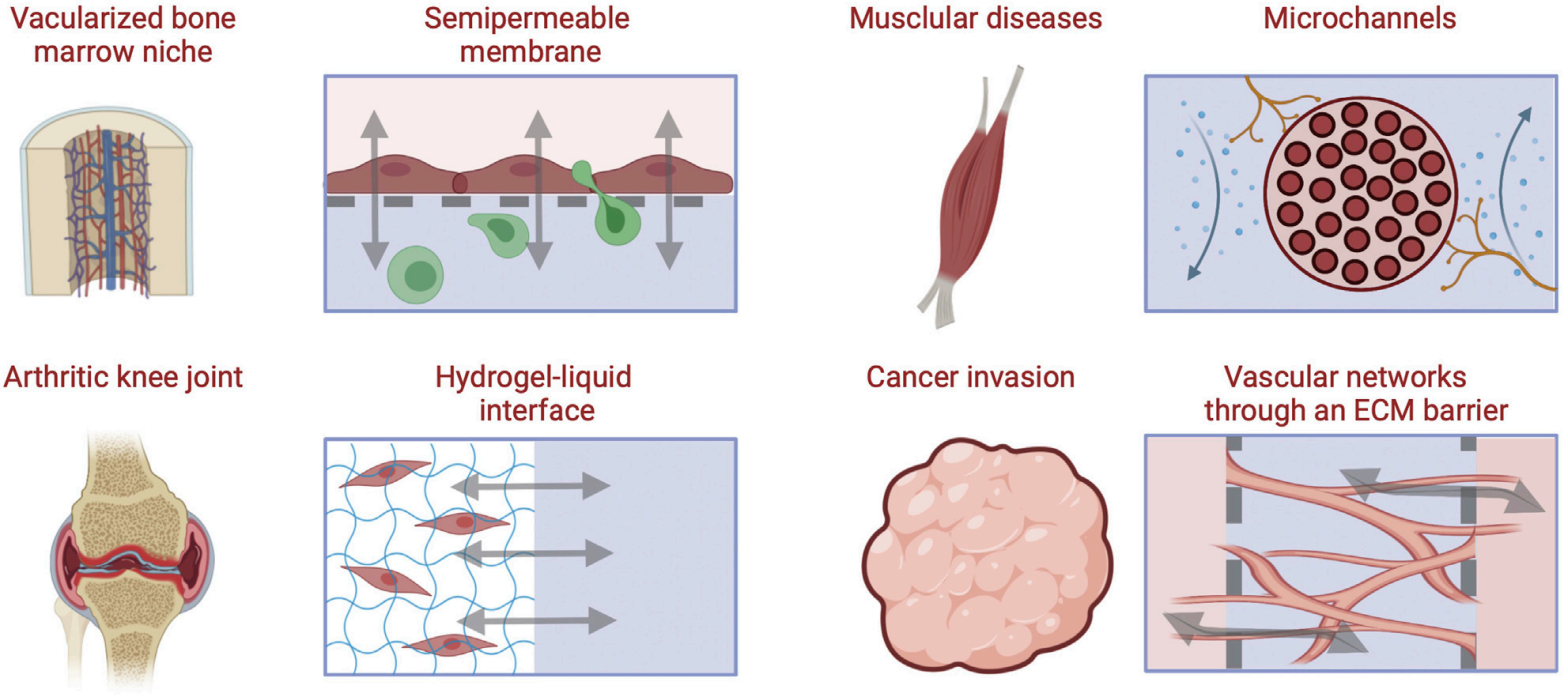
Approaches for engineering barriers and interfaces in musculoskeletal tissues, including porous membrane-based vascular barriers, microchannels, hydrogel-liquid interface, and perfusable microvascular channels network within through an ECM hydrogel barrier [Inspired from (Yeste et al., 2018)].

The following section describes compartmentalized approaches used to engineer biological and vascular barriers in MPS with application examples in musculoskeletal disease models (Figure 5).

#### 5.1.1 Semipermeable Membranes

Early strategies to polarize apical and basal epithelial or endothelial cell surfaces were accomplished with permeable substrates. Membrane filters were used to culture epithelial cells and form polarized monolayers with transport and permeability qualities of *in vivo* transporting epithelium (Cereijido et al., 1978). These models served as experimental platforms still in use today to elucidate epithelial and endothelial disease mechanisms in different organs (Boccellato et al., 2019). Semipermeable membranes separate chambers into two compartments allowing molecular and cellular transport only through nano- to micro-sized pores, respectively. These can be of particular importance in musculoskeletal tissues as fluid flow is incorporated to simulate vasculature, synovial fluid, or interstitial flow and cell cultures are required to be in suspension or direct contact with other cells or tissues. Semipermeable membranes permit exact compartmentalization, often difficult to achieve with ECM scaffold approaches, while allowing for cell-cell signaling Thermoplastics, elastomers, and inorganic materials have been used to fabricate porous membranes. The advantages of permeable membranes in MPS are their biocompatibility, optical transparency, and flexibility. Nevertheless, there are other variables to consider when implementing porous membranes including pore size, mechanical properties (stiffness), and thinness which have been extensively reviewed by Chung et al. (Chung et al., 2018). Compartmentalizing MPS using membranes allows for permeability assays to be performed as supernatants can be collected from both apical and basolateral compartments, delineating biological cues specific to luminal and basal cell surfaces. Additionally, the inclusion of micron-scale pores allows for cell transmigration studies between compartments, while strictly nanoporous membranes (pores < 100 nm) limit communication between tissues to paracrine signaling.

As an example, a recently published joint-on-chip model includes vascularized synovium and articular cartilage PDMS compartments separated by lined trapezoidal posts 90 µm apart creating micropores for cellular transmigration (Mondadori et al., 2021). Monocytes were introduced into the vascularized channel ± TNF-α and shear stress activation and monitored their transmigration into the osteoarthritic (OA) synovial fluid and cartilage compartments, identifying higher transmigration in the presence of OA synovial fluid compared to control medium alone. When monocytes were incubated with CCR2 and CCR5 receptor antagonists a significant reduction of extravasation was observed comparable to the control group where no OA synovial fluid was present. Such models serve as tools to study mechanisms responsible for abnormal macrophage infiltration and can be expanded to other leukocytes to study inflammatory musculoskeletal diseases. One limitation of many of the microfabricated membranes lies in their supraphysiologic thickness which slows the exchange of soluble factors between compartments but can negatively impact longitudinal microscopic imaging. Therefore, technologies to fabricate ultrathin porous membranes, which would mimic the *in vivo* barriers is a priority in MPS fabrication research.

#### 5.1.2 Hydrogel-Liquid Interface

Hydrogels physically support cells in MPS while enabling direct interaction with surrounding fluid and/or other tissues. When incorporating hydrogels into MPS, a vital property to consider in addition to cytocompatibility and mechanical properties are molecular diffusion rates. Liquid interfaces with optimized hydrogels are powerful instruments to mimic native joints, simulate local inflammation, and administer therapeutics for drug screening assays. Additionally, such hydrogels can be patterned, and 3D printed to create musculoskeletal models with precisely controlled architectures. As an example, an osteochondral-tissue chip using human iPSCs was developed to model the pathology of OA by embedding induced mesenchymal progenitor cells (iMPC) in a methacrylated gelatin hydrogel. The cell-laden construct was placed in a bioreactor with two separated fluidic channels accessing the top and bottom of the construct respectively. A chondrogenic medium was perfused in the top channel and an osteogenic medium was supplied through the bottom conduit over 28 days to induce chondrogenic/osteogenic differentiation of the iMPCs. The construct was shown to effectively model OA in the cartilage compartment through the introduction of IL-1β, and responds by reducing inflammatory cytokines when treated with Celecoxib, a COX-2 inhibitor commonly used as a first-line treatment for OA (Lin et al., 2019).

#### 5.1.3 Microchannels

Directly patterning microchannels into the MPS substrate is an effective approach to control the cellular architecture and organization. Microchannels link two adjacent chambers and can be lined with monolayer forming cells such as endothelial or epithelial cells to completely cover the inner surfaces or they can be used to guide axonal growth. Microchannels have been used to model the human neuromuscular junction (NMJ) transmission upon exposure to inhibitors, where motoneurons (MNs) can communicate with skeletal muscle cells in two separate compartments connected by microchannels embedded in a PDMS BioMEM construct. Physiological behavior was evidenced in the system as high frequency excitation of the MNs drove the myotubes to contract into tetanus while pharmacological NMJ inhibitors added to the muscle compartment demonstrated that MN-induced muscle contraction could be attenuated (Santhanam et al., 2018). Such a strategy can be pivotal in studies of neuromuscular degenerative diseases or injury states.

#### 5.1.4 Vascular Networks Embedded in ECM

Perfusable channels are often designed to be embedded in hydrogel constructs within MPS, usually to form vascular networks. Vascular networks are critical to many diseases such as metastatic cancer, inflammation, and fibrosis. Incorporating vasculature into musculoskeletal MPS also permits immune cell components to be introduced and drug studies to be tested through vascularized models which can provide estimates of PK/PD. Vascular networks for MPS have been successfully incorporated into various tissues and organs and showed improved outcomes for disease modeling and physiological function (Jeon et al., 2015; Chen et al., 2017; Ewald et al., 2021). Vascularization in MPS can be achieved by physically patterning hollow channels through hydrogels and then infusing vascular cells to adhere to the tunnel walls forming vasculature with a perfusable lumen. Additionally, 3D printing techniques can be used to form more complex constructs such as larger blood vessels (Dellaquila et al., 2021). Nonetheless, these architectures can be difficult to achieve in MPS dimensions and often do not recapitulate the isotropic architecture of *in vivo* vasculature, such as in the bone marrow, a substantial challenge in musculoskeletal tissues that must be overcome to develop physiologically relevant MPS models.

Another approach to address this challenge is the self-assembled formation of vasculature from endothelial lined microchannels through a gel-liquid interface with angiogenic gradients driving vascular growth in the hydrogel (Kim et al., 2013). These mature networks have been shown to form impermeable vascular barriers allowing for perfusion of immune cells, biochemical signaling, and treatments between connected compartments (Ewald et al., 2021). For example, MPS were developed to model 3D vascularized muscle fibers using a sequential molding technique using optogenetic, Channelrhodopsin-2 expressing muscle fiber bundles and 3D vessels (600 μm diameter) in a collagen gel (Osaki et al., 2018). The interaction between the muscle fibers and the endothelial cells was modulated through secreted angiopoietin-1 and optical stimulation of muscle tissue contraction, which induced angiogenic sprouting. On the other hand, myogenesis and improved muscle contraction were regulated by interactions with the endothelial cells through angiopoetin-1/neuregulin-1 signaling, demonstrating the feasibility of embedding vascular networks in hydrogel models of musculoskeletal tissue, and the importance of accounting for signaling between endothelial cells and myocytes in the formation of functional muscle models.

### 5.2 Engineered Heterogenous Tissue Interfaces

**FIGURE 6:**
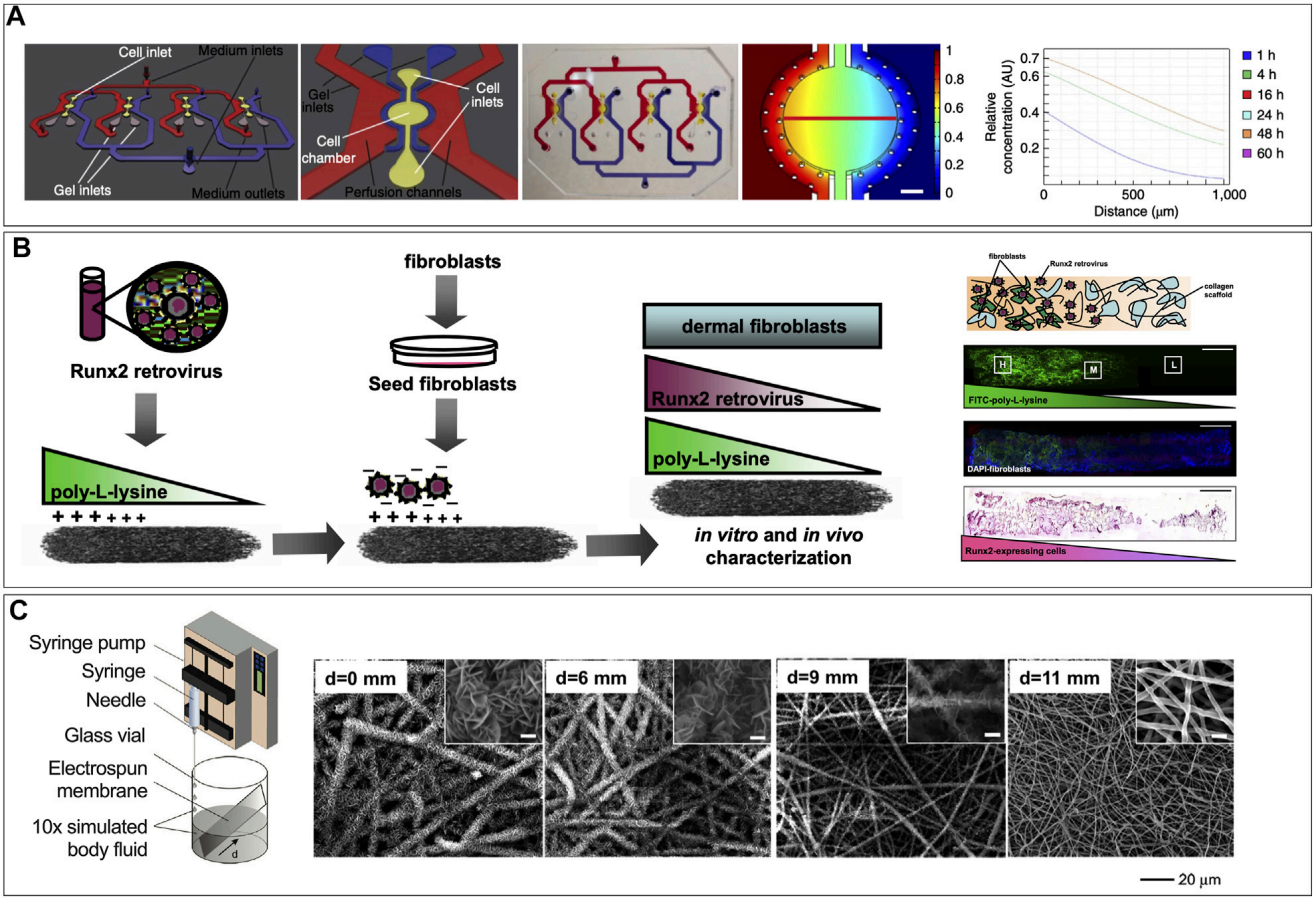
Strategies for creating gradients that could be implemented in microphysiological systems. **(A)** Engineered signaling centers for the spatially controlled patterning of human pluripotent stem cells, showing schematic of the microfluidic device a single unit of the device, a picture of the PDMS microfluidic device filled with colored ink in the distinct compartments, and computational simulation of the diffusion of a reference molecule from the source side of the cell chamber after 48 h of perfusion. [Reproduced from (Manfrin et al., 2019) with permission]. **(B)** Schematic representation of scaffold-mediated spatially graded gene delivery strategy implemented to create a spatial distribution of Runx2 retrovirus within 3D matrices. The proximal portion of collagen scaffolds was coated with PLL before incubation in retroviral supernatant and fibroblast seeding. Representation of a fibroblast-seeded construct containing spatial patterns of noncovalently immobilized retrovirus, showing distribution of Runx2 retrovirus (R2RV) created by partially coating the proximal portion (left side) of collagen scaffolds with PLL at a dipping speed of 170 μm/s before incubation in retroviral supernatant and cell seeding. Confocal microscopy images demonstrating a graded distribution of FITC-labeled PLL (green) **(B)** and FITC-labeled PLL gradient colocalized with uniformly distributed cell nuclei (DAPI, blue), and immunohistochemical staining for eGFP (pink) counterstained with hematoxylin (blue) revealed elevated eGFP expression on the proximal scaffold portion coated with PLL-R2RV. [Reproduced from (Phillips et al., 2008. Copyright (2008) National Academy of Sciences, United States) with permission]. **(C)** Nanofiber scaffolds with gradations in mineral content for mimicking the enthesis using a graded coating of calcium phosphate on a nonwoven mat of electrospun nanofibers by submerging in 10× concentrated simulated body fluid added at a constant rate to linearly reduce the deposition time from the bottom to the top end of the substrate (d refers to the distance from the bottom edge of the substrate) (Left). SEM images of graded calcium phosphate coatings on the PLGA nanofibers from different regions, with d = 0 mm representing the longest exposure to SBF and d = 11 mm representing the shortest exposure to SBF [Reproduced from (Li et al., 2009) with permission].

In musculoskeletal tissues, the integration of soft-to-hard interfaces *in vitro* such as the cartilage-bone junction, neuromuscular junctions, myotendinous (muscle-to-bone) junction, and entheses (tendon-to-bone junction) into MPS are significant unmet needs. Modeling injury to biological junctions in a humanized model requires the integration of multiple tissues and phases into one MPS. This can be a particularly challenging process *in vitro* where cell matrix needs differ widely. Rowland et al. (2018) developed an anatomically shaped cartilage derived matrix construct to spatially organize chondrogenic and osteogenic differentiation of a bone marrow-derived mesenchymal stem cell population by controlling the site-specific expression of transcription and growth factors (Rowland et al., 2018). This model relies on the antagonistic effects of chondrogenic and osteogenic growth factors in a co-culture organoid model, which was designed to investigate aberrant inflammation in joints (Rowland et al., 2018). The cellular and structural complexity at interface regions connecting muscle to tendon (myotendinous junction or MTJ) or tendon/ligament to bone (enthesis) represent important functional adaptations and are commons sites of acute and chronic injuries. The largely extracellular tendon aspect of the MTJ develops ridge-like protrusions, which interdigitate with finger-like myofibrils in the muscle to increase contact surface area and enhance the MTJ strength (Knudsen et al., 2015). The engineering feat of recreating the interdigitating interface between muscle fibers and the mostly acellular ECM of tendon remains unmet, although recent scaffold-free approaches are showing promise (De Pieri et al., 2021). The enthesis is a common feature of both tendon and ligament insertion into bone. Recent evidence revealed continuous, spatially graded mineralization of the collagenous tendon as it inserts into the bone (Patel et al., 2018). The discovery of the graded composition inspired novel approaches for interface tissue engineering via biomimetic gradation of mineralization in nanofibrous-scaffolds (Li et al., 2009). The engineering of these heterogenous tissue interfaces remains an unmet need and a challenge for MPS models of MTJ or enthesis injury, but several strategies have been described in the literature (Phillips et al., 2008; Li et al., 2009; Manfrin et al., 2019). Although not specific to soft-to-hard interfaces, modeling heterogenous tissues requires the optimization of co-culture conditions for the several cell types involved. This presents unique hurdles which could potentially be overcome by incorporating gradient generators within the chips control the spatial concentrations of differentiating factors (Figure 6A) (Manfrin et al., 2019). Alternatively, scaffold-mediated spatially graded gene delivery strategy implemented to create a spatial gradient of Runx2 retrovirus within 3D matrices has been implemented as a tissue engineering approach for the enthesis (Figure 6B), which could be translated in MPS (Phillips et al., 2008). Additionally, mimicking the enthesis using a spatially graded coating of calcium phosphate on electrospun nanofibers can be a viable strategy for modeling the enthesis in a MPS (Figure 6C) (Li et al., 2009). Furthermore, substrate interactions such as stiffness, topography and pore density, which can affect cell differentiation must also be factored in.

### 5.3 Incorporation of Immune Cells and Inflammatory Factors

Inflammation represents the body’s response to cell and tissue damage from harmful agents or injury. The initiation, progression, and resolution phases of inflammation in musculoskeletal tissues and disease are important considerations in developing effective treatments. Acute injury to musculoskeletal tissues initiates the recruitment of inflammatory cells (neutrophils, monocytes, lymphocytes, and mast cells) to the injury site. Neutrophils, the first phagosomes recruited, set the stage to the activation of monocytes and lymphocytes to attack and eliminate foreign organisms and agents. The functional consequences of activation of circulating monocytes to macrophages, which represent the innate immune response, depend on their polarization, with M1 macrophages effecting phagocytosis and proinflammatory cytokines secretion and M2 macrophages typically credited with anti-inflammatory cytokine and growth factor secretion to initiate tissue repair (Sunwoo et al., 2020). Lymphocytes represent the body’s adaptive immune system and complement the innate immune responses. Among these are T and B cells, which are varied with complex effector functions both promoting and attenuating inflammatory responses (Murphy and Weaver, 2016).

The need to incorporate immune system function on MPS is not unique to those interested in musculoskeletal research. However, efforts to create “immune-system-on-a-chip” and similar platforms have largely been limited to three types of systems. These include systems with immune cells in tumors, systems investigating the interaction between endothelium and immune cells, and systems modeling the inflammatory process as a whole (Polini et al., 2019). While these models are not specific to musculoskeletal diseases, they provide valuable insights. An example of this is the three organ “body-on-a-chip” model used to study monocyte activation using an MPS model to recapitulate the complex responses of the immune system to various stimuli (Sasserath et al., 2020). As described earlier, the activation of monocytes to macrophages is a key component of many musculoskeletal diseases. Consequently, macrophage plasticity makes it difficult to study macrophages and their niches *in vivo.* For example, the intrinsic vs. extrinsic factors affecting the fate, heterogeneity and plasticity of synovial macrophages is still largely unknown, yet breakthroughs in this area can dramatically improve joint treatments (Culemann et al., 2019; Hannemann et al., 2021). Therefore, information gained from these types of models will be valuable in informing future musculoskeletal therapies. Targeted musculoskeletal MPS systems that also incorporate immune and inflammatory components are emerging. As discussed earlier, a microfluidic model of the articular joint has been used to study monocyte extravasation in osteoarthritis (Mondadori et al., 2021). Another example is the bone-marrow-on-a-chip which allows for the study of hematopoietic physiology and effect of toxicity on immune cells in different disease models (Chou et al., 2020). These examples and others serve to highlight the potential for musculoskeletal MPS models to incorporate immune system components and the continued motivation for prioritizing this research.

### 5.4 Biomechanical Actuation and Loading

When considering the application of MPS to the musculoskeletal system, much of the utility of MPS relies on the ability to integrate mechanical stimulation through various actuation strategies. The functions of mechanical stimulation during development are well established, including in the musculoskeletal system (Mammoto et al., 2013). In development, forces exerted by muscles on neighboring tissues are important regulators of proper formation of mature musculoskeletal tissues. These effects are diverse and include controlling the 3-dimensional geometry of bones, developing strong muscle-tendon junctions, and many others. Even beyond development, mechanical forces have been shown to contribute to the health and function of mature tissues as well as aging ones. One example that has been studied extensively is the potential compensation of age-related bone loss by mechanical stimulation (Shwartz et al., 2013). Additionally, preclinical and clinical studies have demonstrated the effectiveness of physical therapy (mechanical loading) as a component of treatment protocol after musculoskeletal injuries. Therefore, it is imperative that musculoskeletal MPS incorporate mechanical stimuli that are native to the tissue.

**FIGURE 7:**
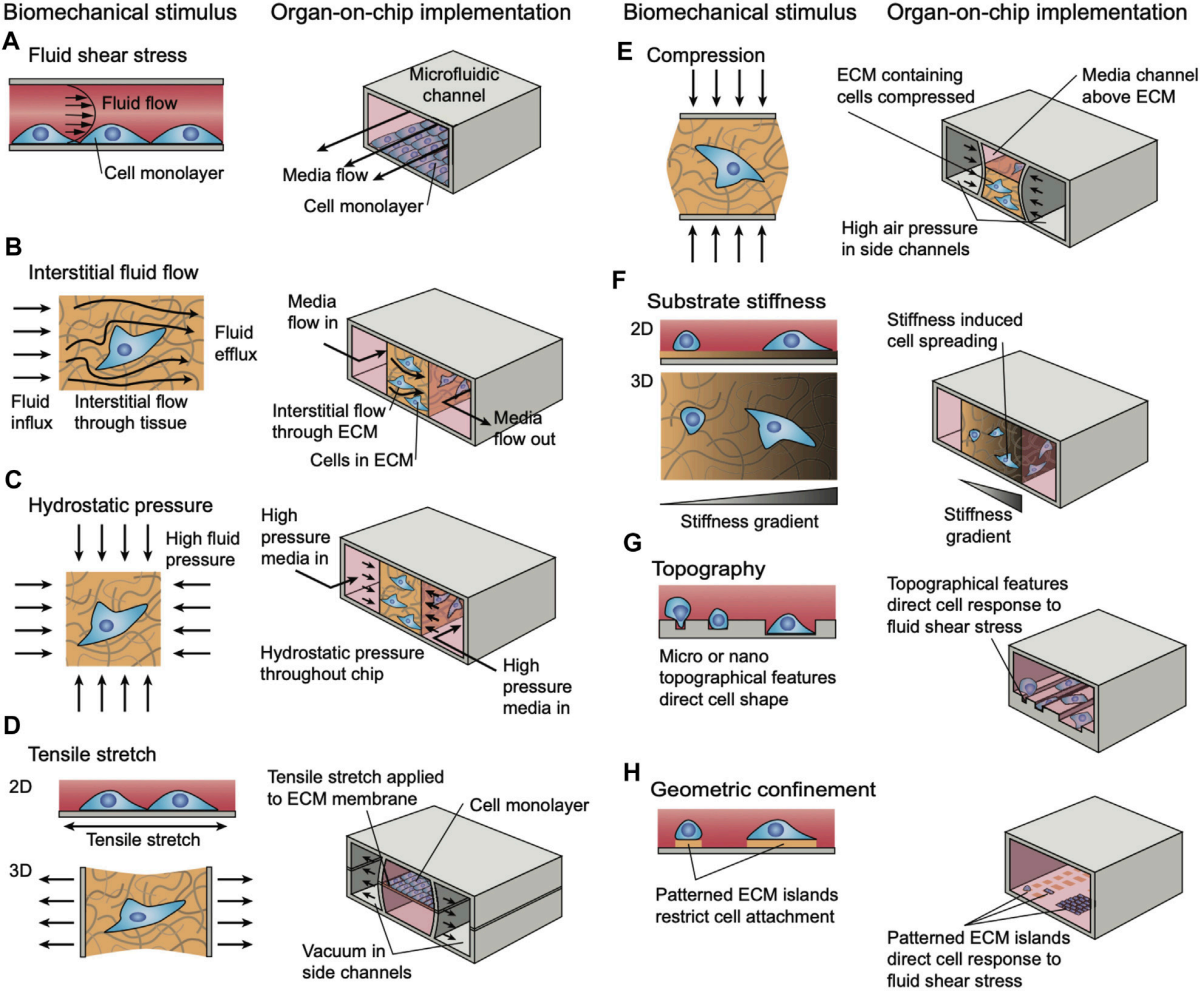
Strategies for simulating different modalities of biomechanical loading and stimuli in microphysiological systems. Reproduced from (Thompson et al., 2020) under the Creative Commons Attribution License (CC BY).

There are numerous strategies of mechanical actuation applied in tissue-on-a-chip research platforms. However, implementation in musculoskeletal MPS models is still in development. One of the most common ways to mechanically stimulate MPS models is to deform the substrate upon which cells or tissues are cultured. Often, this is accomplished through the use of vacuum chambers to stretch a flexible PDMS membrane, as has been reported in a model alveolar-capillary membrane (Huh et al., 2010). A similar technique has been implemented in a muscle model of muscular dystrophy in an MPS (Michielin et al., 2015) to stretch microtissues made of fibroblast-laden collagen hydrogels (Walker et al., 2018). In these cases, the substrate actuates 2D monolayers through membrane deflection, to simulate peristalsis or breathing motions, and 3D tissues which typically actuate multi-axially throughout the volume. These principles have been applied to MPS of cartilage-on-a-chip to introduce gradients of compression (Lee et al., 2018; Paggi et al., 2020) to simulate movements of joints. Other techniques involve using mechanical actuators to physically manipulate a substrate or device (Kamble et al., 2016) are frequently used for multi-axial actuation, however these techniques will often require larger experimental set-ups outside of the microfluidic device to provide the necessary actuation. These techniques may also be adapted to provide compressive loads on cells and tissues, for example to study osteogenesis from several stem cell sources (Park et al., 2012). Other, more passive, techniques for incorporating mechanical stimuli in MPS models through direct device manipulation include constraining cells in specially designed compartments and patterned substrates that illicit phenotypic changes (Männik et al., 2021). In general, these techniques are used in elastomeric PDMS-based microfluidic devices (Figure 7) (Thompson et al., 2020).

As most MPS models rely heavily on microfluidics for proper function, many have utilized controlled fluid flow to provide mechanical stimulation in their devices (Ergir et al., 2018). Typically, the stimulation by fluid flow is used to study the effects of shear stress in biological systems. This has been extensively utilized in recent research including in several bone models (Middleton et al., 2017). Still, fluidic-based actuation is most reasonable for systems modeling environments with fluid flow, such as blood vessels.

Beyond these two main categories of modeling *in vivo* forces and deformations, other, less common, techniques are also described in the literature (Ergir et al., 2018). These include acoustic based actuator, electromechanical and electromagnetic, and optical techniques. Electromechanical and electromagnetic techniques may involve electrical stimulation of cells in the device or utilizing the electromagnetic properties of biomolecules or magnetic beads. Optical techniques may include the use of optical tweezers to provide small-scale stimulation of MPS models. Many of these techniques are not common presently as research in the field is still rapidly progressing.

### 5.5 Afferent Nociceptive Signaling (Pain) Outcomes

**FIGURE 8:**
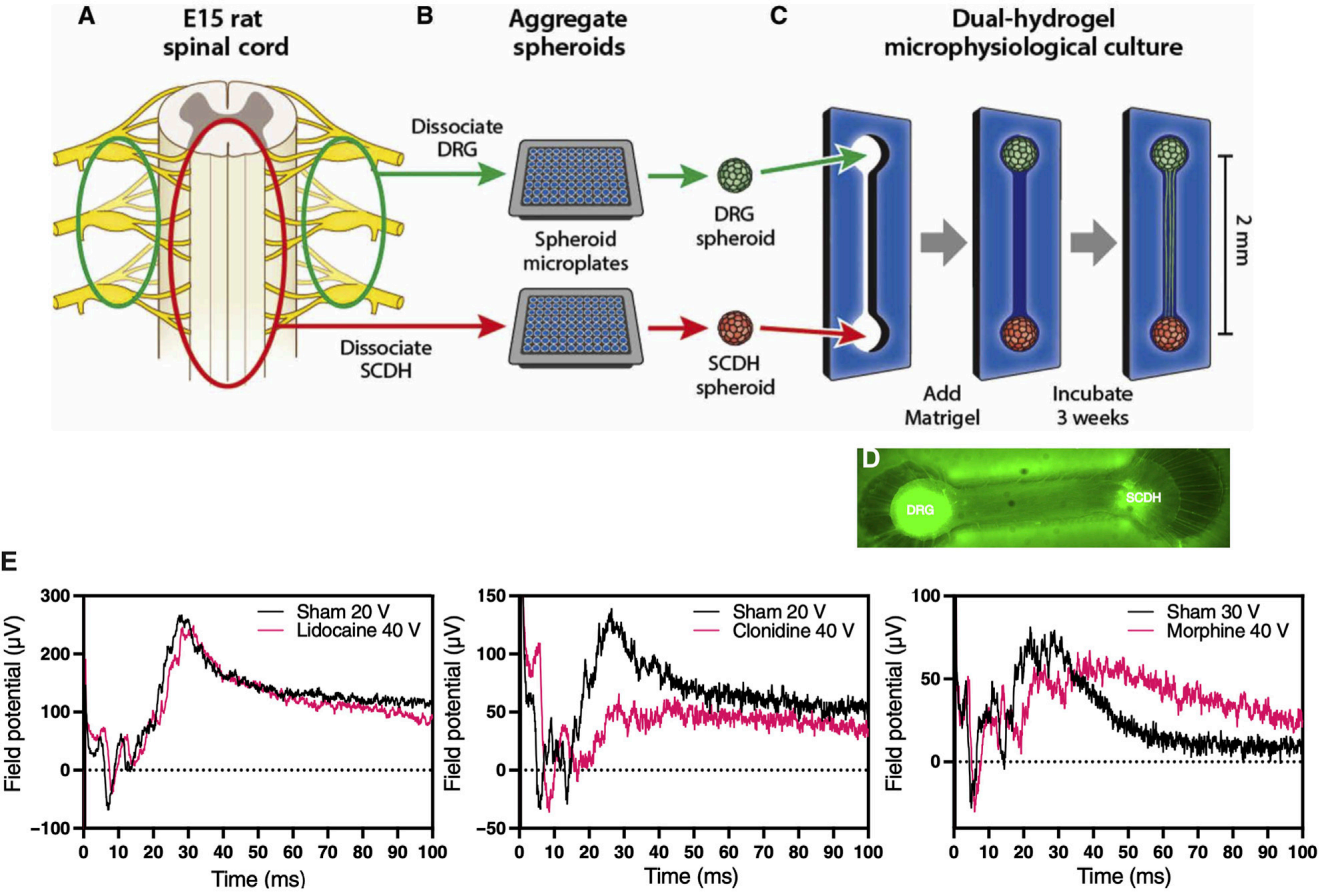
Morphine-sensitive synaptic transmission in a microphysiological model of afferent nociceptive signaling. **(A)** Dorsal root ganglion (DRG) (green) and spinal cord dorsal horn (SCDH) (red) nerve tissues are harvested from E15 rat embryos. **(B)** Tissue is pooled by type, dissociated into a single-cell suspension, and aggregated in spheroid microplates to generate a batch of spheroids identical in size and composition. **(C)** A growth-restrictive outer-gel polyethylene glycol mold is fabricated to shape the cultures; spheroids are seeded in the mold, and the mold is filled with growth-permissive Matrigel. Over 3 weeks of culture, microphysiological tissue emerges **(D)** from which system-level functional data are obtained. **(E)** Differential desensitization of the afferent DRG input through treatments with lidocaine (left), clonidine (middle), and morphine (right) traces. [Reproduced from (Pollard et al., 2021) with permission].

Peripheral sensory neurons known as nociceptors are responsible for relaying pain perception to specialized centers in the brain. Many acute and chronic musculoskeletal pathologies are painful. In fact, pain is often times linked to musculoskeletal functional impairments, and as such represents a key patient reported outcome in clinical trials, which is very challenging to replicate in MPS-based virtual clinical trials. Several MPS are being developed to model efferent nociceptive signaling in an effort to screen experimental compounds for analgesic effect that could alleviate the need for using opioids. A recent study modeled spinal cord dorsal horn, a common target for analgesic intervention, by coculturing peripheral and dorsal spinal cord nerve cells in a MPS, which led to autonomous emergence of native nerve tissue macrostructure and distinct synaptic transmission in response to different analgesics, including morphine, lidocaine, and clonidine (Figure 8) (Pollard et al., 2021). This demonstrates the potential to incorporate nociceptors in MPS to record surrogate signals for pain. To that end, Moy et al. (2021) incorporated sensory neurons into an established microJoint MPS to monitor the interplay between the peripheral nervous system and joint tissues (Moy et al., 2021). This could be accomplished by incorporating microchannels to enable sensory neuron innervation of joint tissues, where the neural activity is assessed with micro electrode arrays (MEAs) and fura2-based calcium imaging. Using a 3D printed PDMS system, the sensory dorsal root ganglion (DRG) neurons were allowed to extend in the microchannels and exposed to conditioned media from OA-modeled microJoint. This led to an increase in calcium flux in the sensory neuron microchannels. While the work is preliminary, it indicates the feasibility to incorporate sensory neurons in MPS to study OA pain via recording the electrical activity of the neurons or a surrogate calcium signal, which could be extended to other musculoskeletal pathologies.

### 5.6 Integration of Sensors

In addition to close recapitulation of tissue physiology and disease biomarkers *in vitro*, it is highly desirable for tissue chip models to be able to sense various parameters of the system to understand the dynamics of healthy tissue physiology, as well as in disease or injury states. Current MPS employ numerous sensing modalities, including fluorescence microscopy (Zahavi et al., 2015; Sheyn et al., 2019), electrical resistance of tissue barriers (Henry et al., 2017), microelectrode arrays for electrically active tissue (Jeong et al., 2018) and electrochemical signaling (Ezra et al., 2015), pH and dissolved gases (Bavli et al., 2016; Mousavi Shaegh et al., 2016; Prill et al., 2016), and stress/pressure sensors for muscle contractility (Aung et al., 2016), among others. These have revealed a great deal of information for physical and structural parameters of well-controlled MPS.

In addition, protein secretion by damaged or otherwise stimulated cells is an important aspect of musculoskeletal disease and injury. While the sensing mechanisms mentioned above are effective at yielding information about many different physical parameters of the system, the ability to sense proteins secreted by altered cells in real time would increase the value of the information obtained from such a model considerably. Current tissue chip models utilize sensors of primarily imaging outcomes or off-chip measurements of secreted proteins via standard assays. This leaves a great need in the field of musculoskeletal research for tissue chip models that incorporate sensitive, real-time protein sensors for the elucidation of disease and injury mechanisms. Some models include immunosensing modules for secreted analytes (Zhang et al., 2017); however, such models lack sensitivity and are downstream of the MPS, resulting in diluted analytes and loss of temporal resolution. To our knowledge, no MPS features real-time sensing of secreted analytes on the same chip as the cells being studied.

Real-time sensing for specific proteins secreted by an MPS will require integrating sensors able to achieve the sensitivity threshold required to observe relevant quantities of analytes secreted in musculoskeletal injury and disease. Current research indicates the detection of cytokines, as well as other proteins, at levels as low as pg/mL in serum or in macroscale *in vitro* models. However, some microfluidic *in vitro* models have shown single-cell secretion of cytokines from T-cells at levels in the ng/mL range at close proximity to the cells (Li et al., 2018). Thus, serum levels of secreted analytes might not accurately reflect levels in close proximity to their cellular source, due to downstream dilution. By relying on downstream sensor modules, analytes of interest become diluted, and the temporal information about their release may be lost. This further motivates sensor integration and highlights that tissue chip models incorporating label-free biosensing will need significant dynamic range as well as high sensitivity to detect and quantify important analytes.

To improve the quality of information obtained from *in vitro* tissue models, it is important to consider certain design parameters as the field progresses. Disease and injury sequelae can occur on short timescales, including in musculoskeletal injuries. Therefore, it is critical that *in vitro* models of these conditions are able to measure tissue responses in real time and should preferably incorporate inline antibody-functionalized sensors in close physical proximity to the tissue construct. Another important consideration is sensor regeneration, or the restoration of saturated antibody-functionalized surfaces. Many antibody regeneration protocols exist, usually consisting of harsh chemical treatments (Goode et al., 2015). If regeneration is required during experimental timeframes, the microfluidics of the system must be organized so that regeneration solutions do not come into contact with tissue components, since most regeneration formulae are toxic to cells.

### 5.7 Arrayed Formats for High Throughput Screening

While MPS enable gathering high content biochemical, functional, and histological data from each device, their value can be increased when the platform is composed of an array of chips that can be assayed quickly and accurately for high throughput screens. Multiplexing has become common practice for researchers to analyze multiple factors at once, improving data consistency by allowing multiple targets to be investigated within the environment. The physical footprint of MPS must be amenable to high-throughput tests including access to culture media and tissues, visualization of tissue cultures, and simple assembly and operation. For example, a 96-well plate platform for bulk production of human muscle microtissues (hMMTs) for phenotypic drug testing has been developed (Afshar et al., 2020). The fabrication of the tissue plate is a simple one-stage casting step of a 96-well plate, which requires uncomplicated workflows with low number of cells used. Purified CD56^+^ myogenic progenitor cells were differentiated into the skeletal muscle model, which was used to predict structural and functional treatment responses of Dexamethasone, Cerivastatin, and IGF-1, in addition to predicting effects of the chemotherapeutics Gemcitabine and Ironotecan (Afshar et al., 2020). Such models would be simple to integrate into drug screening and toxicity workflows as they can provide large data sets with minimal variability as tissue constructs can be differentiated identically *in situ*. Significant advancements have also been made in the functional genomics screening field where Caft-ID, a CRISPR-based microRaft array technology, followed by gRNA identification, was developed to couple the power of image-based phenotyping of stress granules with easy-to-use pooled CRISPR screening workflows on microRaft arrays. Stress granules are protein-RNA cytoplasmic foci that form transiently during cellular perturbations including oxidative stress, heat shock and immune activation and have been linked to degenerative diseases and even cancer. The Craft-ID platform expands on the power of CRISPR-screening to high-content imaging and machine learning to allow the interrogation of genetic modulators of subcellular and cell-morphological phenotypes which, previously were inaccessible with bulk infection models (Wheeler et al., 2020). This trend towards more automated, miniaturized MPS increases throughput and content of platforms, which has clear advantages for the early stages of drug discovery and screening in terms of improving rigor and reproducibility.

## 6 Conclusion

The poor translation from preclinical animal studies to human clinical applications and incomplete understanding of the mechanisms of action and the lack of biomarkers to define biological efficacy represent significant barriers that impede the development of disease modifying therapies for musculoskeletal conditions. The emerging technology of hMPS might offer transformative opportunities to cost-effectively address the aforementioned barriers. MPS is a wide-encompassing term for sophisticated *in vitro* human models, also known as tissue- and organ-on-a-chip, carefully designed to offer standardized predictive models by mimicking physiologically relevant aspects of living tissues and organ systems. Applications in modeling musculoskeletal acute and chronic injury are slowly developing. Keys to translational implementation MPS models of musculoskeletal pathologies include developing strategies to: engineer vascular and biological barriers and heterogenous tissue interfaces; incorporate immune cells and inflammatory factors; enable biomechanical actuation to simulate *in vivo* loading; incorporate sensory neurons (nociceptors) to record surrogate measurements for pain; integrate inline sensors for real-time monitoring of secreted proteins critical to modulating these dynamic processes; and develop arrayed formats for high throughput screening. Furthermore, issues of scalability, reproducibility, and validation, which are not discussed in this review, are also of paramount importance and have been addressed in other reviews (Hargrove-Grimes et al., 2021).
